# Population Pharmacokinetics of Meropenem and Vaborbactam Based on Data from Noninfected Subjects and Infected Patients

**DOI:** 10.1128/AAC.02606-20

**Published:** 2021-08-17

**Authors:** M. Trang, D. C. Griffith, S. M. Bhavnani, J. S. Loutit, M. N. Dudley, P. G. Ambrose, C. M. Rubino

**Affiliations:** a Institute for Clinical Pharmacodynamics, Inc., Schenectady, New York, USA; b Rempex Pharmaceuticals, San Diego, California, USA

**Keywords:** meropenem, vaborbactam, population pharmacokinetics

## Abstract

Meropenem-vaborbactam is a broad-spectrum carbapenem–beta-lactamase inhibitor combination approved in the United States and Europe to treat patients with complicated urinary tract infections and in Europe for other serious bacterial infections, including hospital-acquired and ventilator-associated pneumonia. Population pharmacokinetic (PK) models were developed to characterize the time course of meropenem and vaborbactam using pooled data from two phase 1 and two phase 3 studies. Multicompartment disposition model structures with linear elimination processes were fit to the data using NONMEM 7.2. Since both drugs are cleared primarily by the kidneys, estimated glomerular filtration rate (eGFR) was evaluated as part of the base structural models. For both agents, a two-compartment model with zero-order input and first-order elimination best described the pharmacokinetic PK data, and a sigmoidal Hill-type equation best described the relationship between renal clearance and eGFR. For meropenem, the following significant covariate relationships were identified: clearance (CL) decreased with increasing age, CL was systematically different in subjects with end-stage renal disease, and all PK parameters increased with increasing weight. For vaborbactam, the following significant covariate relationships were identified: CL increased with increasing height, volume of the central compartment (*V*_c_) increased with increasing body surface area, and CL, *V*_c_, and volume of the peripheral compartment were systematically different between phase 1 noninfected subjects and phase 3 infected patients. Visual predictive checks demonstrated minimal bias, supporting the robustness of the final models. These models were useful for generating individual PK exposures for pharmacokinetic-pharmacodynamic (PK-PD) analyses for efficacy and Monte Carlo simulations to evaluate PK-PD target attainment.

## TEXT

Meropenem is a broad-spectrum carbapenem with *in vitro* activity against Gram-negative bacteria, including *Enterobacterales* and other important pathogens associated with hospital-acquired infections, such as Pseudomonas aeruginosa and anaerobes (1–3). While meropenem is stable against many beta-lactamases, resistance to meropenem and other carbapenems can be mediated by class A serine carbapenemases, especially Klebsiella pneumoniae carbapenemases (KPC) (4). Vaborbactam is a cyclic boronic acid beta-lactamase inhibitor that has broad inhibitory activity against several clinically important beta-lactamases. These include class A carbapenemases such as KPC-2, KPC-3, KPC-4, BKC-1, FRI-1, and SME-2 and class A extended-spectrum beta-lactamases (ESBLs) such as CTX-M, SHV, and TEM. Vaborbactam also has inhibitory activity against class C cephalosporins (e.g., CMY, P99) (5–8). *In vitro* and *in vivo* studies show that meropenem in combination with vaborbactam is highly active against Gram-negative pathogens, including KPC-producing *Enterobacterales* (9, 10).

Meropenem-vaborbactam as a fixed-dose combination (2 g–2 g over 3 h every 8 h [q8h] with dose adjustments for renal impairment) was approved by the U.S. Food and Drug Administration for the treatment of patients with complicated urinary tract infections, including pyelonephritis (11). The European Medicines Agency approved the same meropenem-vaborbactam dosing regimen with similar dose adjustments for renal impairment for the treatment of patients with hospital-acquired pneumonia (HAP), including ventilator-associated pneumonia (VAP), and those with complicated intra-abdominal and urinary tract (including acute pyelonephritis) infections (cIAI and cUTI, respectively) (12).

As part of the development program, data from two phase 1 and two phase 3 studies conducted to evaluate meropenem-vaborbactam were used to develop population pharmacokinetic (PK) models for meropenem and vaborbactam. Results of population PK analyses are critical to enable a better understanding of the drug disposition in subjects and patients. Use of a population PK model, together with individual PK data from phase 3 studies, also allows for the conduct of pharmacokinetic-pharmacodynamic (PK-PD) analyses to further inform efficacy and potential safety events (13, 14). Finally, a population PK model, together with preclinical PK-PD targets, *in vitro* surveillance data, and Monte Carlo simulation, can be used to confirm late-stage dosing regimens, including for special populations, and supports the decisions for interpretive criteria for *in vitro* susceptibility testing (15). The objectives of the population PK analyses described herein for meropenem and vaborbactam were the following: (i) to develop population PK models to describe the disposition of meropenem and vaborbactam using data from noninfected subjects enrolled in two phase 1 studies (16, 17) and infected patients enrolled in two phase 3 studies (18, 19) and (ii) to identify individual descriptors associated with the interindividual variability (IIV) in meropenem and vaborbactam PK.

## RESULTS

### Pharmacokinetic analysis population.

Summary statistics of baseline descriptors for the analysis population consisting of 110 noninfected subjects and 322 infected patients are presented in Table 1. The percentage of males in the model development population was 45%; ages for all subjects or patients ranged from 18 to 92 years. Phase 1 noninfected subjects from study 501 had a moderate range of weight (56.0 to 94.7 kg), and most of these subjects had normal renal function. Phase 1 noninfected subjects from study 504 had a relatively broad range of weight (58.2 to 143 kg) and renal function. Infected patients from two phase 3 studies, Studies 505 and 506, had a broader range of weight (40.0 to 177 kg) and renal function (estimated glomerular filtration rate [eGFR] ranged from 4.50 to 338 ml/min/1.73 m^2^).

**TABLE 1:** Summary statistics or counts of the subject demographic characteristics of analysis population

Variable	Median (minimum–maximum) value^*a*^				
	Phase 1 studies		Phase 3 studies		Total (*n* = 431)
	Study 501 (*n* = 70)	Study 504 (*n* = 40)	Study 505 (*n* = 272)	Study 506 (*n* = 50)^*b*^	
Age (yr)	24.0 (18.0–50.0)	56.5 (44.0–73.0)	58.0 (18.0–92.0)	64.0 (29.0–88.0)	53.0 (18.0–92.0)
BMI (kg/m^2^)	24.0 (19.7–29.4)	31.3 (21.2–43.7)	26.1 (16.5–53.2)	26.0 (17.1–58.5)	26.1 (16.5–58.5)
BSA (m^2^)	1.91 (1.58–2.20)	2.07 (1.63–2.66)	1.80 (1.35–2.48)	1.86 (1.27–2.83)	1.84 (1.27–2.83)
Height (cm)	175 (159–193)	174 (156–190)	165 (148–192)	168 (145–188)	168 (145–193)
eGFR (ml/min/1.73 m^2^)	117 (81.1–203)	46.4 (4.80–142)	86.9 (12.6–241)	72.1 (4.50–338)	90.1 (4.50–338)
Weight (kg)	74.6 (56.0–94.7)	92.8 (58.2–143)	73.8 (43.8–150)	74.8 (40.0–177)	75.0 (40.0–177)
Gender					
Male	52 (74)	25 (63)	90 (33)	25 (50)	192 (45)
Female	18 (26)	15 (37)	181 (67)	25 (50)	239 (55)

a Values for gender are given as number (%) of subjects.

b Initial data from study 506 consisted of 23 patients. An additional 27 patients were available upon study completion.

### Pharmacokinetic data description and outlier analysis.

Data from two phase 1 studies (Studies 501 and 504), one completed phase 3 study (study 505), and one partially completed phase 3 study (study 506) were available for the purpose of developing an initial population PK model. For meropenem, the initial population PK analysis data set contained 4,172 meropenem plasma concentrations from 91 noninfected subjects and 295 infected patients and 834 urine meropenem concentrations from 84 noninfected subjects. For vaborbactam, the initial population PK analysis data set contained 3,988 vaborbactam plasma concentrations from 93 noninfected subjects and 294 infected patients and 746 urine vaborbactam concentrations from 75 noninfected subjects. Samples which were considered outliers and excluded from the analysis were either unreasonably high or low due to potential errors in sampling, data collection, or assay.

A total of 92 meropenem and 94 vaborbactam concentrations were available from an additional 27 infected patients after the completion of study 506. When combined with the initial data, the final data set contained 4,264 meropenem concentrations from 91 noninfected subjects and 322 infected patients and 4,082 vaborbactam concentrations from 93 noninfected subjects and 321 infected patients.

### Population pharmacokinetic analyses. (i) Development of the initial population pharmacokinetic models.

For both meropenem and vaborbactam, a two-compartment model with zero-order input and first-order elimination best described the plasma and urine concentration-time data. For meropenem, interindividual variability was described for the following parameters using a log-normal parameter distribution: clearance (CL), volume of the central compartment (*V*_c_), and volume of the peripheral compartment (*V*_p_). For vaborbactam, interindividual variability was described for all parameters. Residual variability (RV) for plasma and urine was described using a combined additive plus proportional error model. eGFR was evaluated as a covariate for meropenem and vaborbactam renal clearance (CL_R_) in the base structural model using either a linear, power, or a sigmoidal Hill-type function, each of which was evaluated with an intercept term to account for nonrenal clearance (CL_NR_). The sigmoidal Hill-type function with estimation of an intercept term representing CL_NR_ provided a more accurate characterization of CL due to having a larger drop in objective function (68.6 and 144.8 units lower than the power function for meropenem and vaborbactam, respectively) and explaining more of the interindividual variability in CL than did the other functions and was therefore selected to describe the relationship between CL and eGFR for both agents. These models served as the comparator for the subsequent covariate analyses described below.

### (ii) Covariate analyses.

For meropenem, structural covariate parameters tested included weight on clearance, central volume of distribution, and peripheral volume of distribution, as these were the relationships established in a previously developed population PK model (20). Results of the covariate analysis demonstrated that weight was only significant for *V*_c_ and *V*_p_. Examination of the fit of the model to the data from study 504 indicated that the CL in the majority of noninfected subjects with severe renal impairment or end stage renal disease (ESRD) was being substantially overpredicted. Based on the results of the analysis of the data from study 504 (16), various models were attempted in which CL_NR_ was also allowed to vary with changing eGFR. The best fit to the data was obtained when CL_NR_ was allowed to be systematically lower in subjects with an eGFR of ≤30 ml/min/1.73 m^2^. This relationship was therefore incorporated into the model for meropenem. Delta plots were then checked for any remaining potential covariate relationships. There appeared to be an additional relationship between age and meropenem CL. Age was added to the covariate model for CL and resulted in a significant decrease in the minimum value of the objective function (MVOF). The initial population PK model parameter estimates and standard errors for meropenem are provided in Table S1 in the supplemental material.

For vaborbactam, the covariate screening plots revealed multiple potential relationships between baseline descriptors and primary PK parameters. During the first step of forward selection, incorporating a shift in total clearance for phase 1 noninfected subjects in which subjects are allowed to have systematically higher clearance provided the largest drop in the objective function (50.617 units). Subsequent rounds of forward selection resulted in the inclusion of five additional parameter-covariate relationships: (i) relationship between height in centimeters (HTCM) and CL (drop of 13.5 units), (ii) relationship between body surface area (BSA) and *V*_c_ (drop of 32.9 units), (iii) relationship between BSA and *V*_p_ (drop of 18.3 units), (iv) relationship between study phase and *V*_c_ (drop of 15.5 units), and (v) relationship between study phase and *V*_p_ (drop of 20.2 units).The full model was then subjected to backward elimination with more stringent criteria for retention. Removal of the relationship between BSA and *V*_p_ resulted in an improvement of the fit with an 11.5-unit drop in the MVOF. Thus, the relationship between BSA and *V*_c_ was removed from the model in the first round. In the second round, all remaining relationships resulted in a significant increase in the MVOF and were retained. Thus, the backward elimination process was considered complete. The initial population PK model parameter estimates and standard errors for vaborbactam are provided in Table S2 in the supplemental material.

### (iii) Update of the population pharmacokinetic models.

The population PK models for both meropenem and vaborbactam were updated using the final data from study 506. Fitting of the models to the full data set was successful and resulted in limited increases in the IIV. For meropenem, the weight relationship was applied to all parameters using allometric scaling functions. Forcing the weight coefficients to the allometric values resulted in a significant increase in the MVOF, but it did not substantially affect the individual fits and also resulted in a modest increase in the IIV associated with CL and was therefore retained moving forward. After fitting the full covariance matrix, which resulted in a significant drop in the MVOF, IIV was placed on distributional clearance (CL_d_). This updated model resulted in a significant drop in the MVOF and was chosen as the final model for meropenem.

For vaborbactam, attempts were made to standardize the body size relationships to use weight instead of height or BSA but resulted in model instability. The only modification made was fitting a full covariance matrix, which resulted in a further drop in the MVOF, and was thus chosen as the final model for vaborbactam.

### (iv) Final population pharmacokinetic models.

The final population PK model for both agents was a two-compartment model with zero-order infusion and first-order (linear) elimination. The population PK parameter estimates and associated standard errors for the meropenem and vaborbactam models are provided in Tables 2 and 3, respectively. The precision of the PK parameter estimates based on asymptomatic standard error was high throughout with the exception of IIV on CL_d_ for meropenem (283%). In general, the magnitude of the IIV was relatively modest.

**TABLE 2:** Final meropenem population PK model^*a*^

Parameter	Population mean value		Magnitude of IIV (%CV)		Shrinkage (%)
	Final estimate	%SEM	Final estimate	%SEM	
CL (liters/h)					
CL_NR_ (liters/h)	3.85	0.70			
CL_R, max_ (liters/h)	6.58	3.20	44.5	2.90	2.50
eGFR_50_ (ml/min/1.73 m^2^)	40.0	0.10			
Hill coefficient	1.95	13.9			
*V*_c_ (liters)	17.0	22.9	48.4	23.8	9.20
CL_d_ (liters/h)	1.36	0.10	51.7	282.4	29.1
*V*_p_ (liters)	2.32	0.30	37.7	1.90	31.4
Power coefficient of WTKG on CL	0.75	Fixed			
Power coefficient of WTKG on *V*_c_	1.00	Fixed			
Power coefficient of WTKG on CL_d_	0.75	Fixed			
Power coefficient of WTKG on *V*_p_	1.00	Fixed			
Power coefficient of age on CL	−0.526	16.7			
Proportional shift with renal group on CL_NR_	−0.650	10.1			
					
Plasma residual variability					
Plasma proportional error	0.0423	7.30			11.2
Plasma additive error	0.0204	20.0			
Urine residual variability					
Urine proportional error	0.207	23.9			2.60
Urine additive error	0.0511	369.9			

a eGFR_50_, eGFR value at which CLR is half-maximal; WTKG, weight (kg); SEM, standard error of the mean; CV, coefficient of variation; IIV, interindividual variability.

**TABLE 3:** Final vaborbactam population PK model^*a*^

Parameter	Population mean valule		Magnitude of IIV (%CV)		Shrinkage (%)
	Final estimate	%SEM	Final estimate	%SEM	
CL (liters/h)					
CL_NR_ (liters/h)	0.157	12.7			
CL_R, max_ (liters/h)	8.86	3.50	45.6	5.00	0.30
eGFR_50_ (ml/min/1.73 m^2^)	49.7	3.10			
Hill coefficient	2.25	3.40			
*V*_c_ (liters)	17.1	2.90	39.4	10.1	17.3
CL_d_ (liters/h)	2.75	10.5	34.5	76.1	75.4
*V*_p_ (liters)	1.77	11.0	23.0	39.6	65.5
Power coefficient of HTCM on CL	2.24	22.3			
Proportional shift with Phase on CL	0.517	30.8			
Power coefficient of BSA on *V*_c_	1.50	13.0			
Proportional shift with Phase on *V*_c_	−0.215	42.0			
Proportional shift with Phase on *V*_p_	1.28	22.3			
Plasma residual variability					
Plasma proportional error	0.0372	1.90			11.4
Plasma additive error	0.0287	7.80			11.4
Urine residual variability					
Urine proportional error	0.115	4.20			1.30
Urine additive error	5.46	8.70			1.30

a eGFR_50_, eGFR value at which CLR is half-maximal; SEM, standard error of the mean; CV, coefficient of variation; IIV, interindividual variability.

Standard goodness-of-fit plots showed excellent fits to the data (Fig. S1 and S2). The overall coefficient of determination (*r*^2^) values based on observed versus individual fitted concentrations were 0.777 and 0.849 for meropenem and vaborbactam, respectively. In general, the residual plots showed consistent scatter about zero, indicating that there were no significant biases in the fit of the data across the range of fitted concentrations or over time. These plots demonstrate the adequacy of the model fit across subjects and patients.

### Final model evaluation.

**FIG 1:**
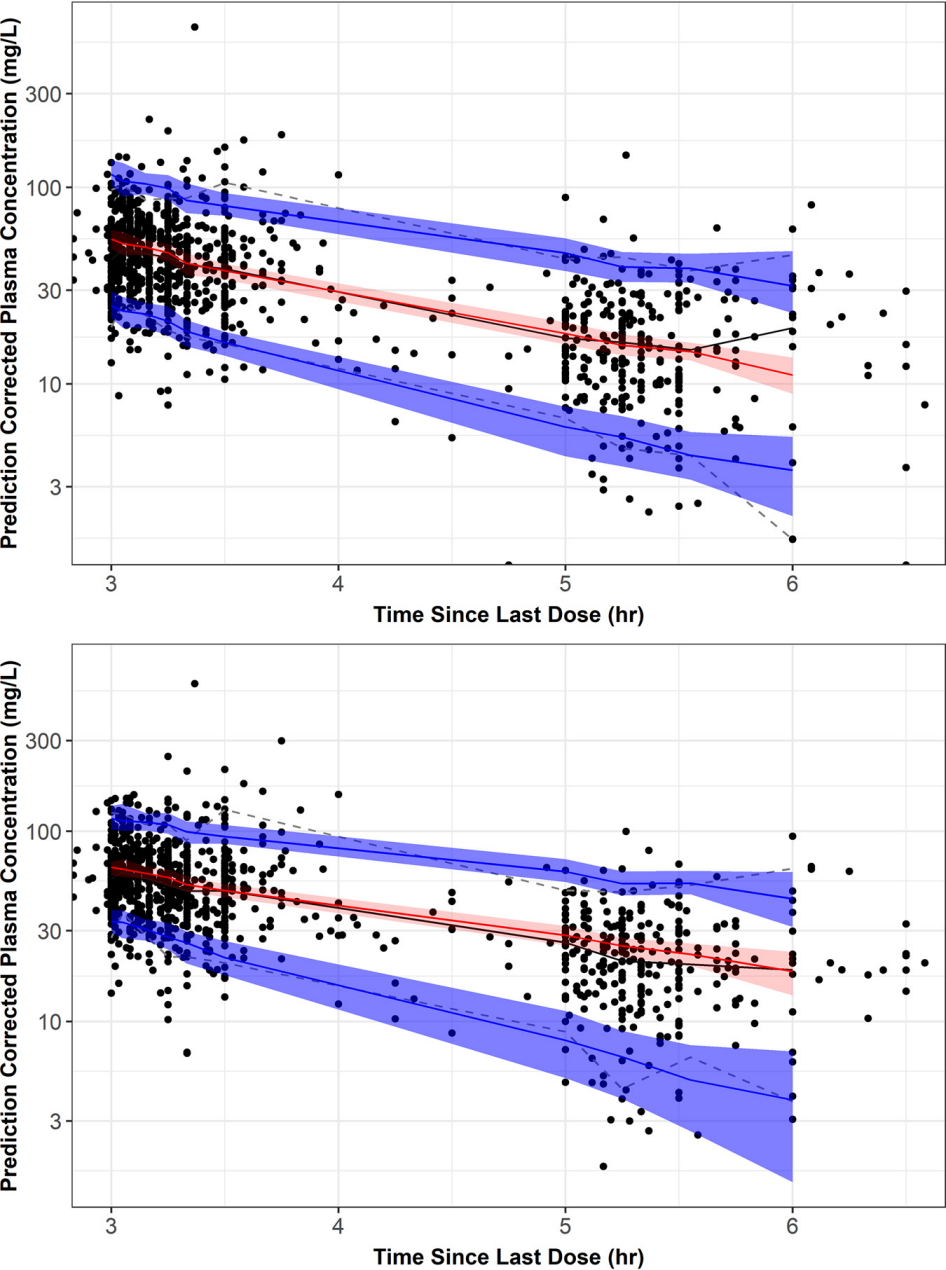
Prediction-corrected visual predictive check plots for the final population PK model for meropenem (top) and vaborbactam (bottom). Circles represent prediction-corrected observed plasma concentrations, while the black lines represent the median (solid line) and 5th and 95th percentiles (dashed lines) of the observed data. The red-shaded region shows the 90% prediction interval for the median simulated values, and the solid red line is the median of the median simulated values. The blue-shaded regions show the 90% prediction intervals for the 5th and 95th percentiles of the simulated values, and the solid blue lines show the median of the 5th and 95th percentiles of the simulated values.

The prediction-corrected visual predictive check (PC-VPC) plots for the meropenem and vaborbactam model are provided in Fig. 1. Overall, the models provided a robust and unbiased fit to the data, demonstrating good alignment between observed concentrations and the model-predicted 5th, 50th, and 95th percentiles.

### Exposures and secondary pharmacokinetic parameter estimates.

Summary statistics for the key PK exposure parameters (maximum concentration [*C*_max_], area under the concentration-time curve over 24 h (AUC_0-24_) on day 1 and at steady-state, the alpha half-life (*t*_1/2__, α_), and the beta half-life (*t*_1/2, β_) are provided in Tables 4 and 5 for meropenem and vaborbactam, respectively.

**TABLE 4:** Summary statistics of key meropenem PK parameters in phase 3 patients receiving meropenem 2 g–vaborbactam 2 g q8h derived from the fit of the updated meropenem population PK model

Parameter	Geometric mean value (geometric %CV)		
	Study 505 (*n* = 272^*b*^)	Study 506 (*n* = 50^*b*^)	Pooled (*n* = 322)
*C*_max_ (μg/ml)^*a*^	52.3 (44.4)	75.4 (55.8)	55.4 (48.4)
Day 1 AUC_0-24_ (μg · h/ml)	564 (49.3)	802 (62.3)	595 (53.3)
Steady-state AUC_0-24_ (μg · h/ml)	548 (49.2)	857 (53.2)^*c*^	586 (52.9)^*c*^
CL (liters/h)	9.61 (57.7)	4.96 (84.4)	8.68 (68.3)
*t*_1/2, α_ (h)	0.751 (23.2)	0.895 (17.1)	0.771 (23.3)
*t*_1/2, β_ (h)	1.79 (30.6)	2.61 (53.7)	1.89 (37.7)

a *C*_max_ represents the highest concentration observed during the first dose interval.

b Based upon protocol-mandated dose adjustment guidelines, 28 patients with renal impairment in study 505 received a dose of meropenem 1 g–vaborbactam 1 g; similarly, nine patients in study 506 received reduced doses of meropenem-vaborbactam due to renal impairment.

c Steady-state AUC_0-24_ estimates were not available for four patients from study 506, as these patients received less than three doses of meropenem-vaborbactam.

**TABLE 5:** Summary statistics of key vaborbactam PK parameters in phase 3 patients receiving meropenem 2 g–vaborbactam 2 g q8h derived from the fit of the updated vaborbactam population PK model

Parameter	Geometric mean value (geometric %CV)		
	Study 505 (*n* = 272^*b*^)	Study 506 (*n* = 49^*b*^)	Pooled (*n* = 322)
*C*_max_ (μg/ml)^*a*^	65.4 (37.5)	90.4 (50.4)	68.7 (41.5)
Day 1 AUC_0-24_ (μg · h/ml)	739 (45.4)	1020 (56.4)	776 (48.7)
Steady-state AUC_0-24_ (μg · h/ml)	710 (51.1)	1190 (70.1)^*c*^	766 (57.9)^*c*^
CL (liters/h)	7.04 (64.4)	3.15 (129)	6.22 (83.1)
*t*_1/2, α_ (h)	0.377 (9.22)	0.390 (6.99)	0.379 (8.99)
*t*_1/2, β_ (h)	1.82 (67.6)	3.87 (112)^*d*^	2.04 (81.5)^*d*^

a *C*_max_ represents the highest concentration observed during the first dose interval.

b Based upon protocol-mandated dose adjustment guidelines, 28 patients with renal impairment in study 505 received a dose of meropenem 1 g–vaborbactam 1 g; similarly, nine patients in study 506 received reduced doses of meropenem-vaborbactam due to renal impairment.

c Steady-state AUC_0-24_ estimates were not available for four patients from study 506, as these patients received less than three doses of meropenem-vaborbactam.

d *t*_1/2, β_ estimates were excluded for two patients from study 506 due to extremely high values (63.5 and 50.9 h, respectively).

**FIG 2:**
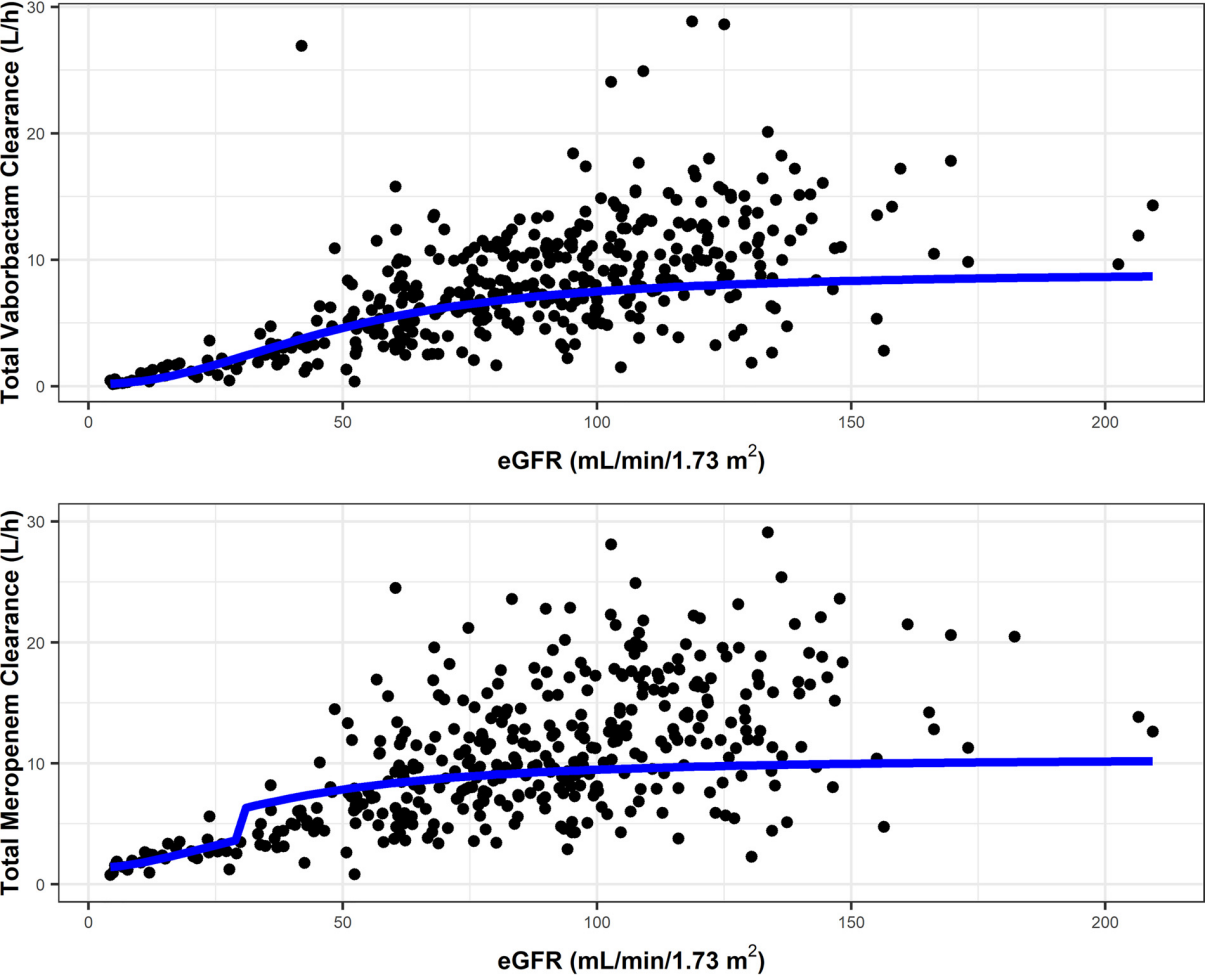
Relationship between individual *post hoc* estimates of CL and eGFR incorporated in the final population PK models for meropenem and vaborbactam. The solid blue line represents the sigmoidal Hill-type function based on final population PK model estimates. With the exception of eGFR, all covariates were set to reference values.

To identify individual descriptors which may have an effect on the exposures of meropenem and vaborbactam, *post hoc* estimates were assessed relative to various covariates. Statistically significant relationships were identified for both meropenem and vaborbactam between clearance and renal function. The effect of renal function on meropenem and vaborbactam concentration-time profiles for a typical simulated infected patient is shown in Fig. S3. Clearance increased in a sigmoidal fashion with increasing eGFR, as shown in Fig. 2. Of note, the shapes of the two relationships were very similar, suggesting that dose adjustments made based upon eGFR for meropenem would allow for appropriate dosing of vaborbactam.

Two different measures of body size were identified as significant covariates in the population PK models for meropenem (weight) and vaborbactam (height and BSA). For meropenem, weight relationships were implemented for all parameters using allometric functions. For vaborbactam, BSA was a significant predictor of the IIV in *V*_c_, and height was a significant predictor of the IIV in CL. For both agents, the resultant impact on the therapeutically relevant parameter of day 1 and steady-state AUC_0-24_ was minimal and indicates that a dose adjustment on the basis of body size is not warranted (Fig. S4).

Given the correlation between age and renal function, it was important to consider potential changes in exposure across age groups relative to eGFR for both meropenem and vaborbactam. There appeared to be no discernible trend for increased exposure in the oldest patients after taking renal function into account (Fig. S5). Similarly, neither gender nor race was expected to have a clinically significant effect on meropenem or vaborbactam exposures (Fig. S6 and S7).

## DISCUSSION

The objectives of the analyses described herein were 2-fold. The first objective was to develop separate population PK models for meropenem and vaborbactam using PK data from noninfected subjects enrolled in two phase 1 studies and PK data from infected patients enrolled in two phase 3 studies. Using these models, the second objective was to identify any individual descriptors associated with IIV in meropenem and vaborbactam population PK parameters, respectively.

The data set used to undertake the population PK analyses for meropenem and vaborbactam described herein, which was based on data for 91 phase 1 noninfected subjects and 322 phase 3 infected patients, was robust. This was evidenced by the broad dose range for each agent (1 to 2 g for meropenem and 0.25 to 2 g for vaborbactam) and eGFR range (4.50 to 338 ml/min/1.73 m^2^) and the large number of plasma and urine concentrations (4,264 and 834 meropenem plasma and urine concentrations, respectively, and 4,082 and 746 vaborbactam plasma and urine concentrations, respectively). The disposition of both meropenem and vaborbactam in noninfected subjects and infected patients was best described by a two-compartment model with linear elimination. The results of the covariate analysis identified several descriptors that were associated with the IIV of meropenem PK. Weight was applied to all PK parameters for meropenem using allometric functions, which is considered standard practice (21). In addition to the impact of renal function on CL_R_, renal impairment was also incorporated as a covariate on CL_NR_, as bias in the fit of the model to the meropenem concentration-time data was observed in noninfected subjects with severe renal impairment or ESRD enrolled in study 504. After inclusion of the relationship between eGFR and CL_NR_, a relationship between age and meropenem CL became evident, which was also included in the final population PK model for meropenem. The current population PK model for meropenem provided a reasonable description of the CL of meropenem across a broad range of eGFR values in both noninfected subjects and infected patients.

Although the PK of meropenem has been explored extensively, the above-described data set that was used for this analysis is likely the most robust data set evaluated to date. Importantly, this data set contained 322 infected patients, the majority of which contributed at least three PK samples to the analysis. The robustness of the data set is important to consider when comparing the population mean meropenem CL in patients with normal renal function based on this analysis (about 10 liters/h in a 58-year-old patient with an eGFR of 100 ml/min/1.73 m^2^) with those estimates previously reported. Results of previous population PK analyses for meropenem indicated that the population mean CL of meropenem was closer to 14 liters/h (22–25). In contrast to the current analysis, published population PK analyses for meropenem in infected patients were based on relatively small numbers of patients and somewhat limited sampling schemes. Interestingly, the population mean meropenem CL in patients with an eGFR of 40 ml/min/1.73 m^2^ based on the current analysis was approximately 7 liters/h, a finding that is consistent with other studies in which the PK of meropenem was quantified in subjects with renal impairment (26–28). In addition, the population mean fraction of CL that is renally cleared in patients with an eGFR of 100 ml/min/1.73 m^2^ based on the current analysis was approximately 59%, which is within the range of 54.3% to 83% estimated in previous studies (26, 27, 29–37). CL_NR_ decreasing with decreasing renal function was a finding also reported previously in the renal impairment studies (26–28), which may be attributed to renal metabolism, which in turn decreases with decreasing renal function (27, 31). Ultimately, given the robustness of the data from infected patients available for this analysis, it is clear that the CL of meropenem is lower than would have been expected from previous analyses.

Results of the covariate analysis for vaborbactam demonstrated the following statistically significant covariate relationships: study phase with CL, *V*_c_, and *V*_p_, HTCM with CL, and BSA with *V*_c_. The relationships of HTCM with CL and BSA with *V*_c_ were expected given that these two parameters tend to scale to body size. One potential explanation for the significance of the relationships between study phase and CL, *V*_c_, and *V*_p_ is the difference in PK sampling (intensive in subjects and informative but relatively sparse in infected patients). Ultimately, the fit of the model to the informative data from phase 3 patients suggests that the estimates of CL are robust in the population of interest.

Given that the majority of noninfected subjects and infected patients included in the analysis received meropenem and vaborbactam concomitantly, the distribution of demographics evaluated for the two sets of analyses were similar. Covariate analyses for each drug resulted in the identification of relationships between renal function (eGFR) and CL for both meropenem and vaborbactam. Given that meropenem is cleared primarily by the kidneys (3, 16, 17), it was not surprising that eGFR was a strong predictor of total CL of meropenem. Results of noncompartmental analyses of the phase 1 studies, Studies 501 and 504, demonstrated that both meropenem and vaborbactam were cleared primarily by the kidneys (16, 17). Overall, the relationships modeled for CL across the range of eGFR values were similar for both agents. This finding suggests that relative CL of meropenem and vaborbactam is consistent regardless of eGFR and supports harmonized dose reductions for both agents in patients with reduced renal function (11, 12). The effects of the remaining covariates in the final population PK models for meropenem and vaborbactam were not of sufficient magnitude to warrant dose adjustments.

Population PK models for meropenem and vaborbactam were refined using a pooled data set used to develop the original model and the additional data from infected patients from study 506 after study completion. Updates to the meropenem population PK model included applying allometric scaling, incorporating IIV on CL_d_, and using a full covariance matrix. The final parameter estimates were comparable to the original parameter estimates, and thus, the impact of the model refinements on meropenem exposures is negligible. Updates to the vaborbactam population PK model only included using a full covariance matrix. The final parameter estimates were comparable to the original parameter estimates, with the exception of the proportional shift with phase on CL (0.517 to 0.264) and on *V*_p_ (1.28 to 1.78). Despite these differences, an assessment of the impact demonstrated that the effect on predicted vaborbactam exposures was not impressive. Simulations of typical infected patients demonstrated an increase in AUC of 13% for the final model relative to the initial population PK model following intravenous (i.v.) administration of a 2,000-mg vaborbactam dose on day 1 and at steady-state conditions.

In conclusion, the excellent individual fits obtained using population PK methods indicated that the primary objective of the analysis was met. A robust description of the plasma PK of both meropenem and vaborbactam in the infected patients studied was achieved, such that the derived measures of meropenem and vaborbactam exposure would be expected to be both accurate and precise. The results of covariate analyses for each drug, which demonstrated the influence of eGFR on CL, provided support for adjustments of dose for renal impairment for both meropenem and vaborbactam. The findings of these analyses were useful for the subsequent execution of clinical PK-PD analyses and Monte Carlo simulations to carry out PK-PD target attainment analyses to support meropenem-vaborbactam dose selection and interpretive criteria for *in vitro* susceptibility testing (38, 39). Results of such analyses, the foundation of which was the population PK models described herein, served to provide data to support the regulatory approval of meropenem-vaborbactam in the United States (11) and European Union for the indications granted, including in the latter region for indications such as cIAI and HAP/VAP (12), which were not directly studied.

## MATERIALS AND METHODS

### Study designs.

Data for these analyses were obtained from two phase 1 studies, study 501 and study 504, pooled with two phase 3 studies, study 505 and study 506 (16–19). A brief description of each study is provided below. A summary of dosing regimens, sampling strategies, and the number of noninfected subjects or infected patients considered for the population PK analyses by study is provided in Table S3 in the supplemental material.

Study 501 (ClinicalTrials.gov registration no. NCT01897779) (16) was conducted in noninfected subjects who received various combinations of meropenem (1 or 2 g) and/or vaborbactam (0.25, 1, 1.5, or 2 g) as a single i.v. infusion or multiple i.v. infusions. A total of 80 subjects were enrolled into five dose cohorts with each cohort containing four treatment arms. Subjects received single doses on days 1, 2, and 7 and multiple doses on days 8 through 14 using an infusion duration of 3 h. Plasma PK sampling was performed intensively on each day of single-dose administration. For multiple-dose administration, intensive sampling was performed on day 14. Urine PK samples were collected on days 1, 4, 7, and 14.

Study 504 (ClinicalTrials.gov registration no. NCT02020434) (16) was conducted in noninfected subjects as well as subjects with renal impairment categorized as having either mild, moderate, or severe renal impairment or end-stage renal disease. A total of 40 subjects were enrolled and received a single dose of 1 g meropenem and 1 g vaborbactam in combination in a 3-h infusion. Plasma and urine PK sampling was performed intensively.

Study 505 (TANGO I; ClinicalTrials.gov registration no. NCT02166476) (18) was a phase 3 clinical trial conducted in patients with acute pyelonephritis (AP) or complicated urinary tract infections (cUTI). A total of 550 patients were randomly assigned 1:1 to receive either meropenem-vaborbactam (2 g meropenem–2 g vaborbactam) i.v. q8h or piperacillin-tazobactam 4.5 g (piperacillin 4 g–tazobactam 0.5 g) q8h. After a minimum of 15 doses of i.v. therapy, patients could be switched to levofloxacin 500 mg by mouth every 24 h to complete a total treatment course of 10 days. Treatment could be up to 14 days if clinically indicated. Samples were collected on day 1 within 0.5 h and 2 to 3 h after the end of infusion, on day 3, and the day of the end of i.v. therapy within 0.5 h after the end of one of that day’s infusions.

Study 506 (TANGO II; ClinicalTrials.gov registration no. NCT02168946) (19) was a phase 3 multicenter, randomized, open-label study of meropenem-vaborbactam versus the best available therapy (BAT) in the treatment of patients with infections due to confirmed or suspected carbapenem-resistant *Enterobacterales*. Patients with bacteremia, hospital-acquired/ventilator-associated bacterial pneumonia, and complicated intra-abdominal and urinary tract infections (including acute pyelonephritis) were eligible for enrollment. A total of 77 patients were randomly assigned 2:1 (meropenem-vaborbactam:BAT). Samples were collected for PK analysis on day 1 within 0.5 h and 2 to 3 h after the end of the first infusion and on days 3 and 5 at 0.5 h after the end of one of that day’s infusions. The development of the population PK model was initiated before study 506 was completed. Model development was conducted using a data set of 27 patients who had completed therapy with meropenem-vaborbactam. The model was then refined once the final data set became available.

### Drug concentration assay.

Plasma and urine samples were assayed for meropenem or vaborbactam concentrations using a validated liquid chromatography-tandem mass spectrometry assay at MicroConstants, Inc. (San Diego, CA, USA). The calibration range of the assay for both agents was 0.02 to 100 mg/liter. Samples that were expected to be outside of the validated range were appropriately diluted using blank biological fluid prior to sample analysis.

### Demographics.

Demographic and disease characteristics were used to characterize the analysis population and to evaluate their ability to explain a portion of IIV for selected PK parameters. eGFR was calculated from serum creatinine, age, and gender using the Modification of Diet in Renal Function equation (40). eGFR was calculated at the time of each serum creatinine measurement and was treated as a time-varying covariate for the population PK analyses. During the calculation of eGFR, serum creatinine was also capped at a lower bound of 0.5 mg/dl. Demographic information included age, height, weight, BSA, body mass index (BMI), sex, and race. BSA was calculated using the method of DuBois and DuBois (41). BMI was calculated as weight (in kilograms) divided by height (in meters) squared.

### Handling of outliers and samples assayed as having concentrations below the limit of quantitation.

An outlier was defined as an aberrant observation that substantially deviated from the rest of the observations within an individual. Outliers were excluded owing to the potential for these observations to negatively impact the convergence and/or parameter estimates as noted in the FDA guidance (42).

Plasma concentration values that were below the limit of quantitation (BLQ) were flagged in the data set. The population analysis program then applied the Beal M3 method (43) such that the algorithm considered a BLQ value as a normally distributed, random value somewhere between negative infinity and the limit of quantification. The Beal M3 method maximizes the probability that a concentration observed to be BLQ is also predicted to be BLQ.

### Population pharmacokinetic analyses.

The population PK analyses for meropenem and vaborbactam were conducted using NONMEM software v7.2 (ICON Development Solutions, Ellicott City, MD, USA), implementing the first-order conditional estimation method with interaction. During various stages of model development, population PK models were minimally assessed using the following criteria: (i) evaluation of individual and population mean PK parameter estimates and their precision as measured by the percent standard error of the population mean estimate, (ii) graphical examination of standard diagnostic and population analysis goodness-of-fit plots with possible stratification by various factors such as patient population or dose group, (iii) graphical examination of the agreement between the observed and individual *post hoc* predicted concentration-time data, (iv) reduction in both residual variability and IIV, and (v) comparison of MVOF for nested models or Akaike’s information criterion for nonnested models.

### Development of the initial population pharmacokinetic models.

Since concomitant administration of meropenem and vaborbactam does not affect the plasma or urine PK of either drug (17), separate population PK models were constructed for meropenem and vaborbactam. The population PK model for meropenem was based upon a model that had been previously developed (20). The first step of the PK model development for meropenem involved fitting a two-compartment model (without covariate relationships) to the plasma data for noninfected subjects from study 501 in order to obtain stable priors for the population parameter estimates. For the vaborbactam population PK model, the plasma vaborbactam concentration-time data for noninfected subjects from study 501 was used to determine the initial structure of the model. One-, two-, and three-compartment models with zero-order input and first-order elimination were to be evaluated.

After establishing the most appropriate structural model for both agents, the urinary excretion and plasma concentration data were comodeled to generate estimates of both CL_R_ and CL_NR_. The data for noninfected subjects from study 504 were then incorporated into the data set, and the models were fit to the pooled phase 1 data. Given that both agents are cleared almost exclusively by the kidneys (16, 17), eGFR was evaluated for statistical significance using various functional relationships prior to the formal covariate analysis (i.e., as part of the base structural model). After the identification of an appropriate relationship between eGFR and CL_R_, data for infected patients from study 505 and study 506 were included and the base structural model was fit to the pooled data.

### Covariate analyses.

Several baseline demographic and disease characteristics were evaluated for their impact on the primary PK parameters. The variables evaluated included sex, race, age, weight, height, BSA, and BMI. Covariate exploration involved calculating individual deviations for each parameter by subtracting individual *post hoc* PK parameters from the population mean PK parameter. Plots of the individual deviations for each PK parameter versus each covariate were examined for observable trends and were used to identify an appropriate function to describe the relationship between the PK parameter and the covariate.

Covariate analyses were conducted separately for each agent using stepwise forward selection and backward elimination. Covariates contributing at least a 3.85-unit reduction in the MVOF (α = 0.05, for 1 df) when added to the model univariately were considered statistically significant; only the most statistically significant covariate was added to the model in each step. This process was repeated until no other subject covariates were statistically significant prior to performing a stepwise univariate backward elimination analysis (α = 0.01, for 1 df) to determine the final population PK model for each agent.

### Update of the population pharmacokinetic models.

Given that study 506 was completed after the development of the initial population PK models for both meropenem and vaborbactam (which were used to support the FDA new drug application), these models were subsequently refined by including additional data for infected patients from study 506. The models were refined to improve the fit, the process for which also included attempts to apply allometric scaling and use full covariance matrices. The final models were used to support the marketing authorization application submitted to the European Medicines Agency.

### Final model evaluation.

To assess the ability of the population PK models to reliably describe meropenem and vaborbactam exposure, a PC-VPC was performed using the parameter estimates from the final population PK models. The 5th, 50th, and 95th percentiles of plasma concentrations from simulated noninfected subjects and infected patients were compared with observed data from the phase 1 noninfected subjects and phase 3 infected patients. Due to the heterogeneity in the dosing times across the phase 3 studies, correcting the observed and simulated values to their respective population predicted values allowed for visualization of data in one plot (44).

### Calculation of secondary pharmacokinetic parameters and exposure estimates.

Estimates of *C*_max_ after the first dose, day 1 and steady-state AUC_0-24_, *t*_1/2__, α_, and *t*_1/2, β_ were generated for all phase 3 infected patients included in the population PK analyses by using a simulated PK profile for each patient using the individual *post hoc* PK parameters from the final population PK models and the mrgsolve package in R (45).
