# Supplementary material for: Population Pharmacokinetics of Meropenem and Vaborbactam Based on Data from Noninfected Subjects and Infected Patients
Source: Antimicrob Agents Chemother. 2021 Aug 17;65(9):e02606-20. doi: 10.1128/AAC.02606-20 (PMC8370236; doi:10.1128/AAC.02606-20)
Supplement: Supplemental file 1 — Supplemental material. Download AAC.02606-20-s0001.pdf, PDF file, 0.9 MB [file aac.02606-20-s0001.pdf]

Table S1. Initial meropenem population PK model parameter estimates and standard errors

Parameter	Population mean		Magnitude of interindividual variability (%CV)	
	Final estimate	%SEM	Final estimate	%SEM
CL				
CL_{NR}	3.78	5.60		
CLr,max	6.60	8.60	44.8	15.7
eGFR50	40.8	13.7		
Hill coefficient	1.94	9.90		
Vc	17.4	4.00	44.3	23.5
CLd	1.52	12.6		
Vp	2.50	7.30	11.6	19.9
Power coefficient of WTKG on Vc	0.487	31.8		
Power coefficient of WTKG on Vp	0.324	37.0		
Power coefficient of AGE on CL	-0.430	14.4		
Proportional shift with Renal Group on CL_{NR}	0.349	11.2		
Plasma residual variability				
Plasma proportional error	0.0388	5.60		
Plasma additive error	0.0213	11.2		
Urine residual variability				
Urine proportional error	0.210	19.5		
Urine additive error	0.0575	50.1		

Table S2. Initial vaborbactam population PK model parameter estimates and standard errors

Parameter	Population mean		Magnitude of interindividual variability (%CV)	
	Final estimate	%SEM	Final estimate	%SEM
CL				
CL_{NR}	0.169	12.5		
CLr,max	9.34	3.3	42.4	5.9
eGFR50	47.1	3.0		
Hill coefficient	2.23	3.4		
Vc	16.9	3.9	35.6	12.5
CLd	3.12	8.6	30.8	55
Vp	1.41	27.2	17.5	36.7
Power coefficient of HTCM on CL	2.17	20.6		
Proportional shift with Phase on CL	0.264	43.6		
Power coefficient of BSA on Vc	1.14	18.1		
Proportional shift with Phase on Vc	-0.203	37.3		
Proportional shift with Phase on Vp	1.78	42.2		
Plasma residual variability				
Plasma proportional error	0.035	1.8		
Plasma additive error	0.0236	7.1		
Urine residual variability				
Urine proportional error	0.127	4.3		
Urine additive error	5.97	8.9		

Table S3. Summary of meropenem/vaborbactam clinical studies used in the population PK model analyses

Study Number	Phase	Title	Number of non-infected subjects/ Infected patients	Meopenem/Vaborbactam dosing regimens	Scheduled plasma/urine PK sample collection times[b]
501	1	Phase 1, randomized, double-blind, single- and multiple ascending dose study of meropenem and vaborbactam alone and in combination in healthy adult subjects [1]	80	Groups 1-5: 250 mg/1 g, 1 g/1 g, 1.5 g/1 g, 2 g/1 g, 2 g/2 g<br>Group 6: 2 g/2 g<br><br>Infusion duration: 3 hours (Groups 1-5), 1 hour (Group 6)	Plasma samples after single dose administration: Pre-dose, 1.5, 3, 3.167, 3.5, 3.75, 4, 5, 6, 7, 8, 12, and 24 hours post-dose<br><br>Plasma samples on Days 9, 11 and 13 after multiple dose administration: Pre-dose<br><br>Plasma samples on Day 14 after multiple dose administration: Pre-dose, 1.5, 3, 3.167, 3.5, 3.75, 4, 5, 6, 7, 8, 12, and 24 hours post-dose<br><br>Urine on Days 1, 4, 7, and 14: 0-4, 4-8, 8-12, 12-24, 24-48, and 48-72 post-dose<br><br>Urine on Days 1, 4, and 14: 24-48 post-dose
504	1	An open-label study to evaluate the pharmacokinetics and safety of a single IV dose of meropenem and vaborbactam in healthy adult subjects and subjects with renal insufficiency [2]	41	Single IV infusion of 1 g/1 g<br><br>Infusion duration: 3 hours	Plasma: Pre-dose, 1.5, 3, 3.25, 4, 5, 6, 8, 10, 12, and 24 hours post-dose<br><br>Urine: 0-4, 4-8, 8-12, 12-24, 24-48, and 48-72 post-dose
505	3	A Phase 3 multi-center, double-blind, randomized study in adult patients with complicated urinary tract infections (cUTI) or acute pyelonephritis (AP) [3]	272	CLcr > 50 mL/min: 2 g/2 g q8h<br>CLcr 30-50 mL/min: 1 g/1 g q8h<br><br>Infusion duration: 3 hours	Day 1: 0.5- and 2-3-hours post-infusion<br>Day 3 and last day of IV therapy: 0.5 post-infusion

Table S3. Summary of meropenem/vaborbactam clinical studies used in the population PK model analyses

Study Number	Phase	Title	Number of non-infected subjects/ Infected patients	Meopenem/Vaborbactam dosing regimens	Scheduled plasma/urine PK sample collection times[b]
506	3	A Phase 3 multicenter, randomized, open-label study in the treatment of adults with selected serious infections [4]	50[a]	CLcr > 50 mL/min: 2 g/2 g q8h<br>30< CLcr <50 mL/min: 1 g/1 g q8h<br>20< CLcr <30 mL/min: 1 g/1 g q12h<br>10< CLcr <20 mL/min: 0.5 g/0.5 g q12h<br>CLcr <10 mL/min: 0.5 g/0.5 g q24h<br><br>Infusion duration: 3 hours	Day 1: 0.5- and 2-3-hours post-infusion<br>Days 3 and 5: 0.5 post-infusion

Note: AP, acute pyelonephritis; cUTI, complicated urinary tract infections; h, hour; IV, intravenous; mg, milligrams; PK, pharmacokinetics; q8h, every 8 hours; q12h, every 12 hours; q24h, every 24 hours.

a. PK data from Study 506 consisted of 23 patients and was available for development of the initial models. Upon completion of Study 506, PK data from an additional 27 patients was available for development of the final models.

b. Represents the number of non-infected subjects or infected patients considered for the PK population analysis, which included those who received at least one dose of meropenem and/or vaborbactam and had PK data available. This count by study included any non-infected subjects or infected patients with outlier samples or samples with concentrations below the limit of quantitation and those with any other missing information that were subsequently evaluated for exclusion.

Figure S1. Goodness of fit plot for final meropenem population PK model

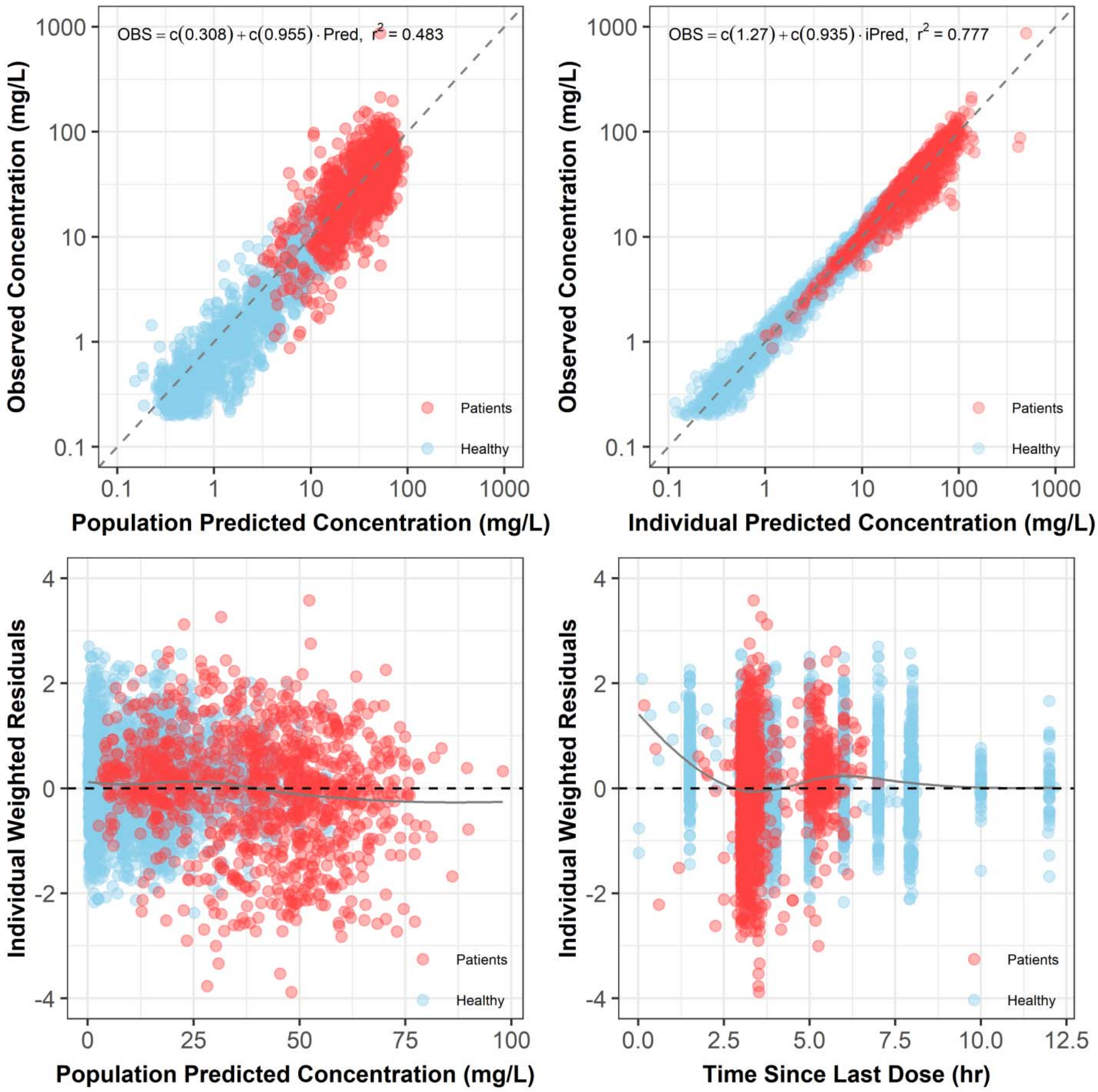

Figure S2. Goodness of fit plot for final vaborbactam population PK model

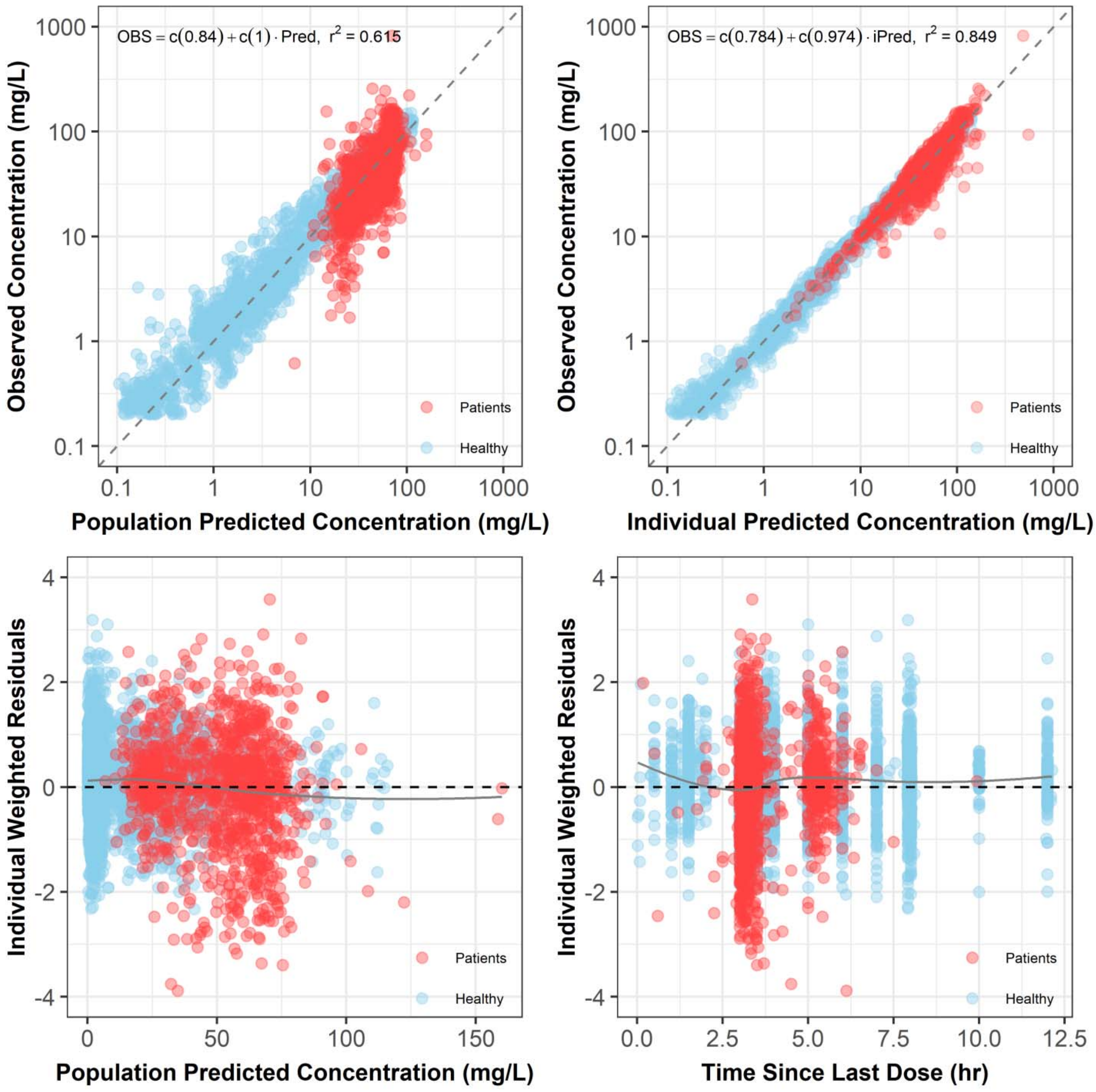

Figure S3. Effect of renal function on meropenem and vaborbactam concentration-time profiles for a typical simulated infected patient

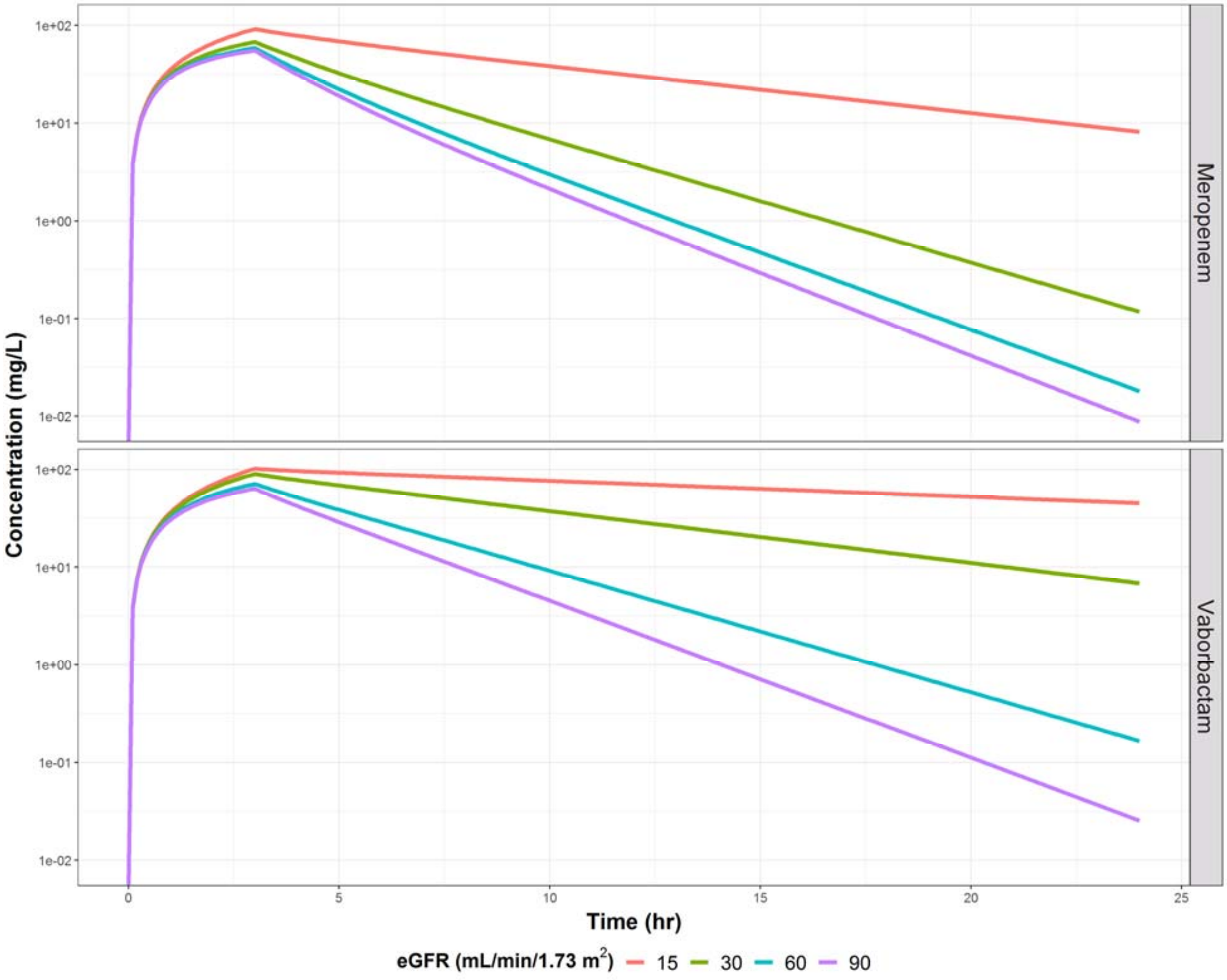

Figure S4. Scatterplot of Bayesian post-hoc AUC_{0-24} versus BSA, height, and weight for Phase 3 infected patients

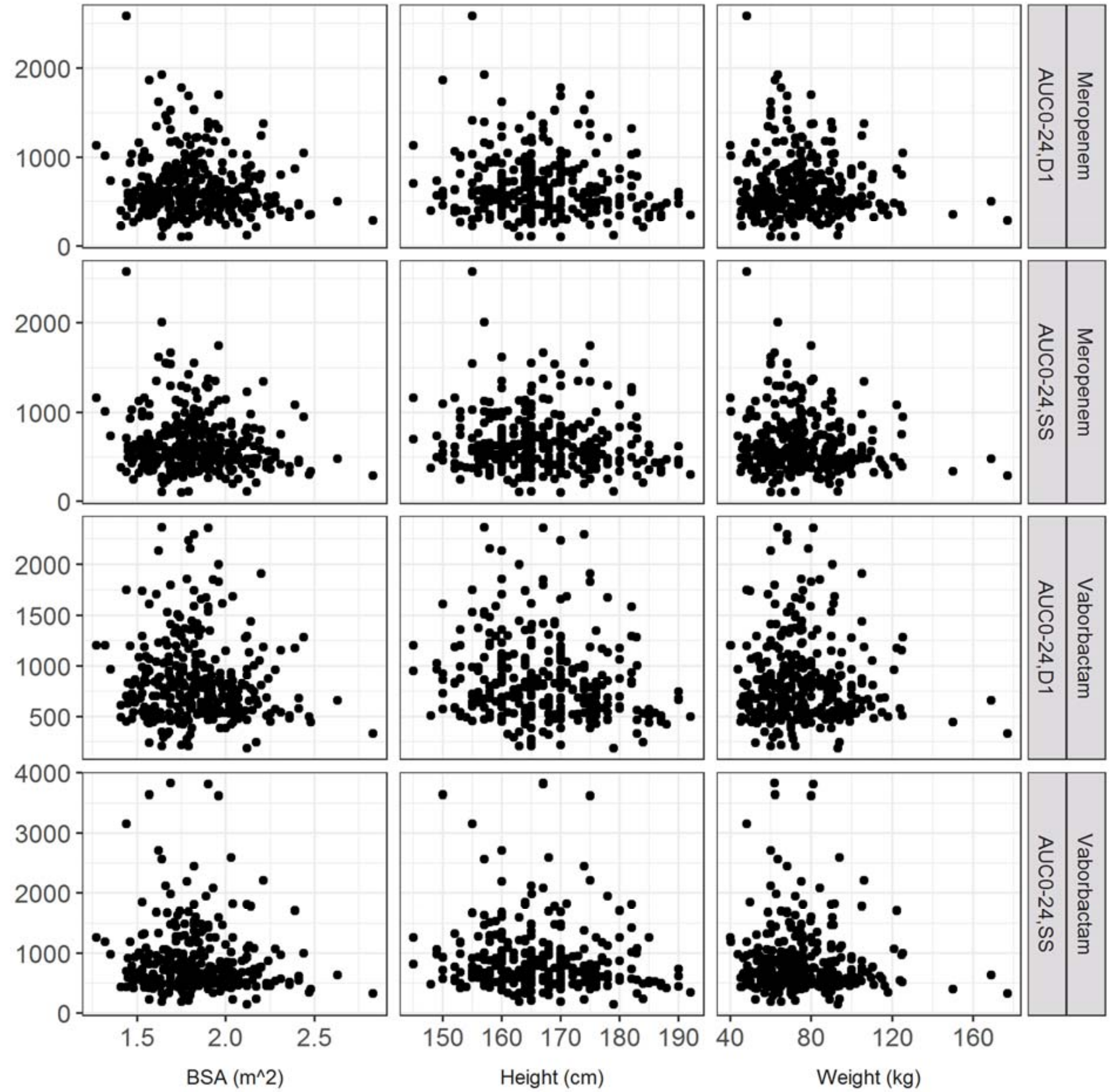

Figure S5. Scatterplot of Bayesian post-hoc AUC_{0-24} versus eGFR, stratified by age category (18-49 yr, 50-65 yr, and ≥66 yr) for Phase 3 infected patients

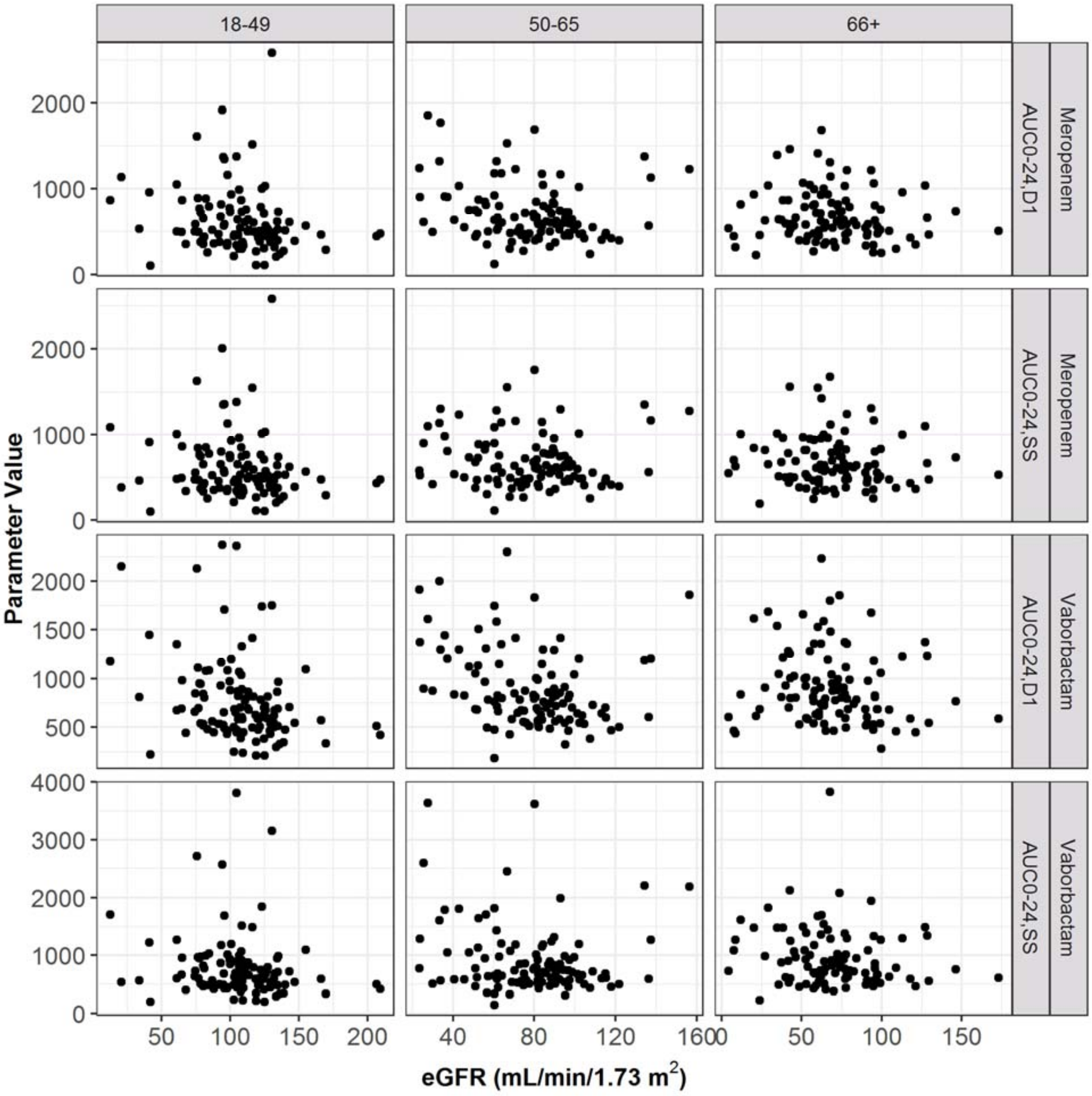

Figure S6. Box-and-whisker plots of the post-hoc AUC_{0-24} estimates for meropenem and vaborbactam in patients enrolled in the Phase 3 studies, stratified by sex

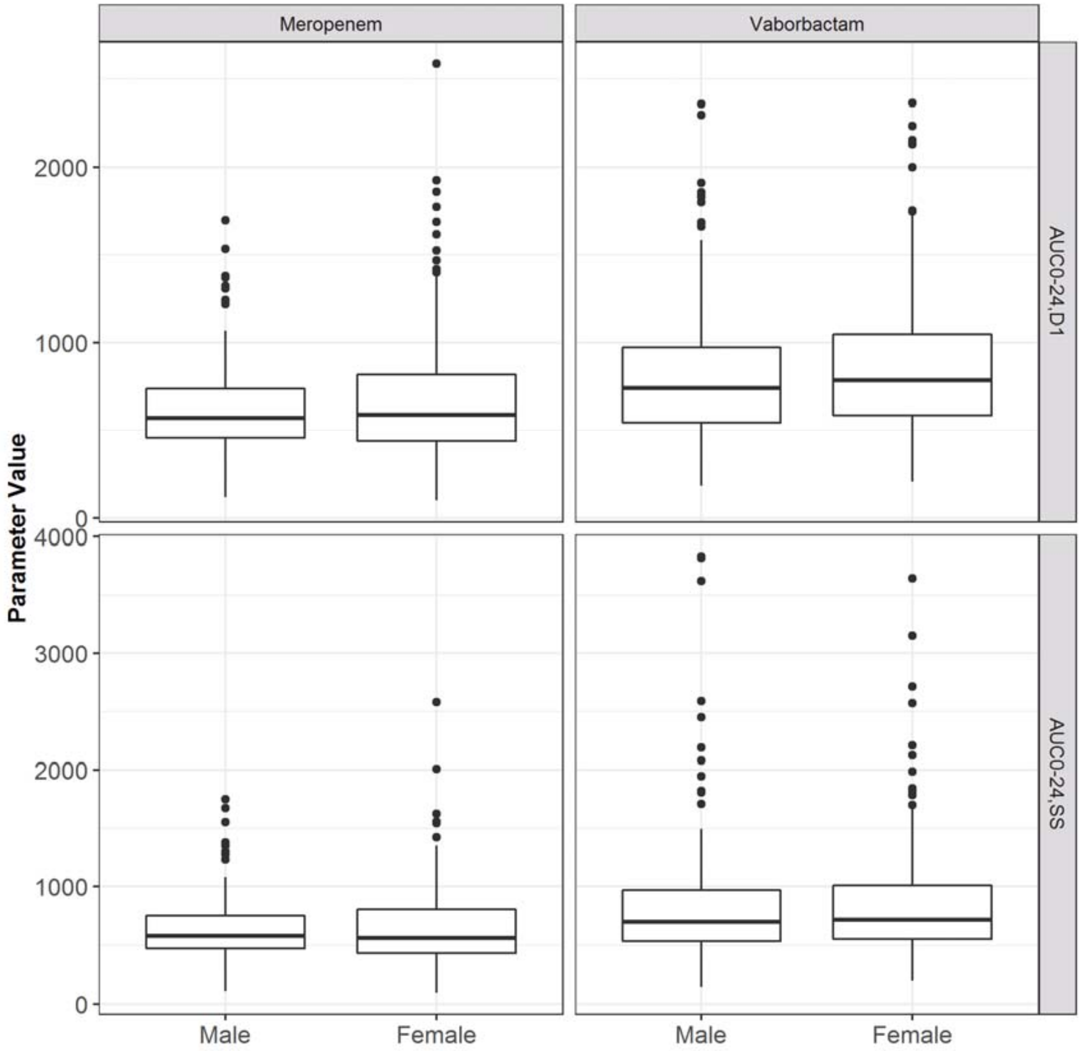

Figure S7. Box-and-whisker plots of the post-hoc AUC_{0-24} estimates for meropenem and vaborbactam in patients enrolled in the Phase 3 studies, stratified by race

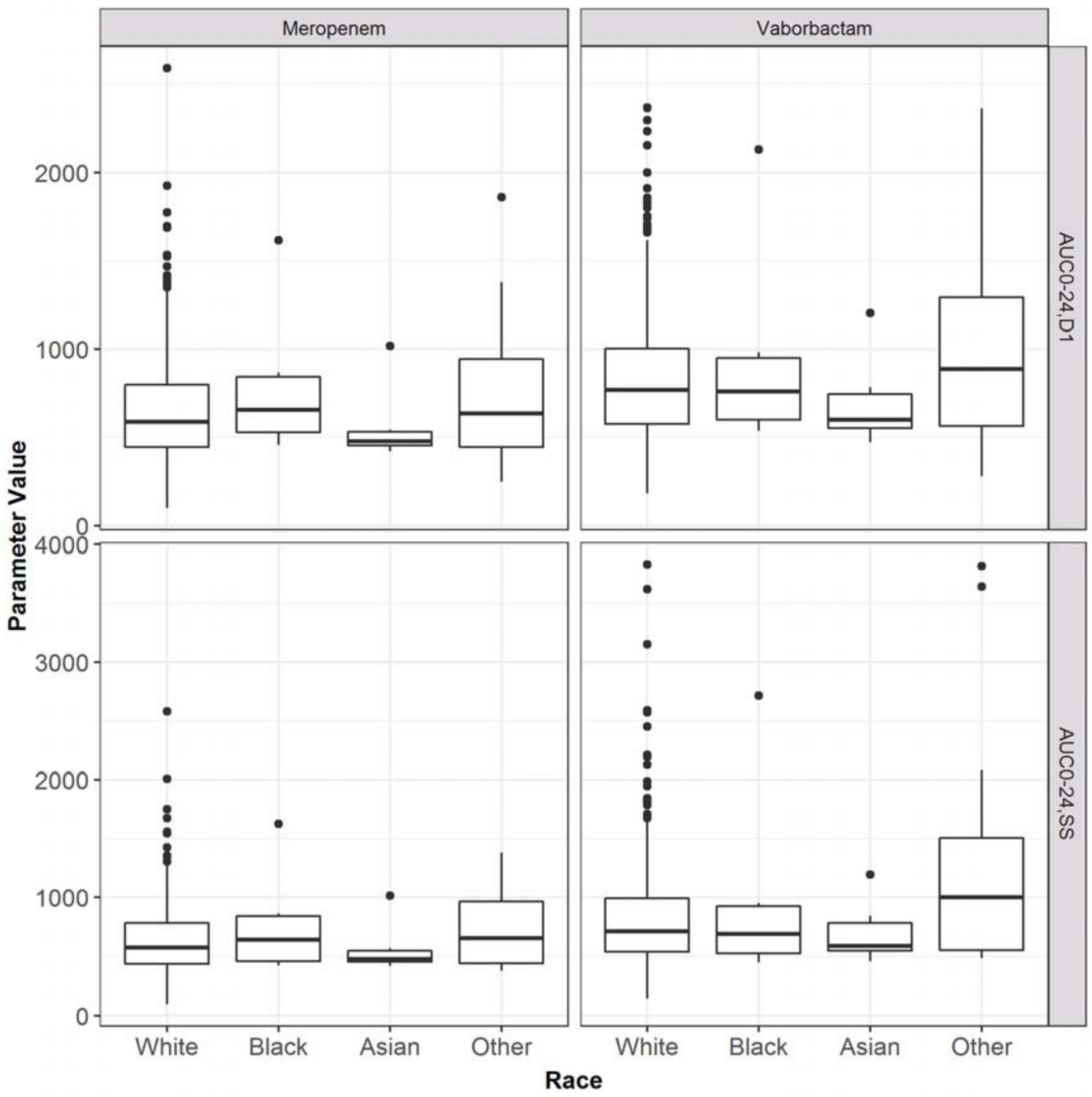

REFERENCES

1. Castanheira M, Huband MD, Mendes RE, Flamm RK. 2017. Meropenem-vaborbactam tested against contemporary Gram-Negative isolates collected worldwide during 2014, including carbapenem-resistant, KPC-producing, multidrug-resistant, and extensively drug-resistant enterobacteriaceae. Antimicrob Agents Chemother 61:e00567-17.

2. Pfaller MA, Huband MD, Mendes RE, Flamm RK, Castanheira M. 2018. *In vitro* activity of meropenem/vaborbactam and characterisation of carbapenem resistance mechanisms among carbapenem-resistant Enterobacteriaceae from the 2015 meropenem/vaborbactam surveillance programme. Int J Antimicrob Agents 52:144-150.

3. Pfizer, meropenem (MERREM IV)® package insert. New York, NY; 2019.

4. Drawz SM, Bonomo RA. 2010. Three decades of β-lactamase inhibitors. Clin Microbial Rev 23:160-201.
